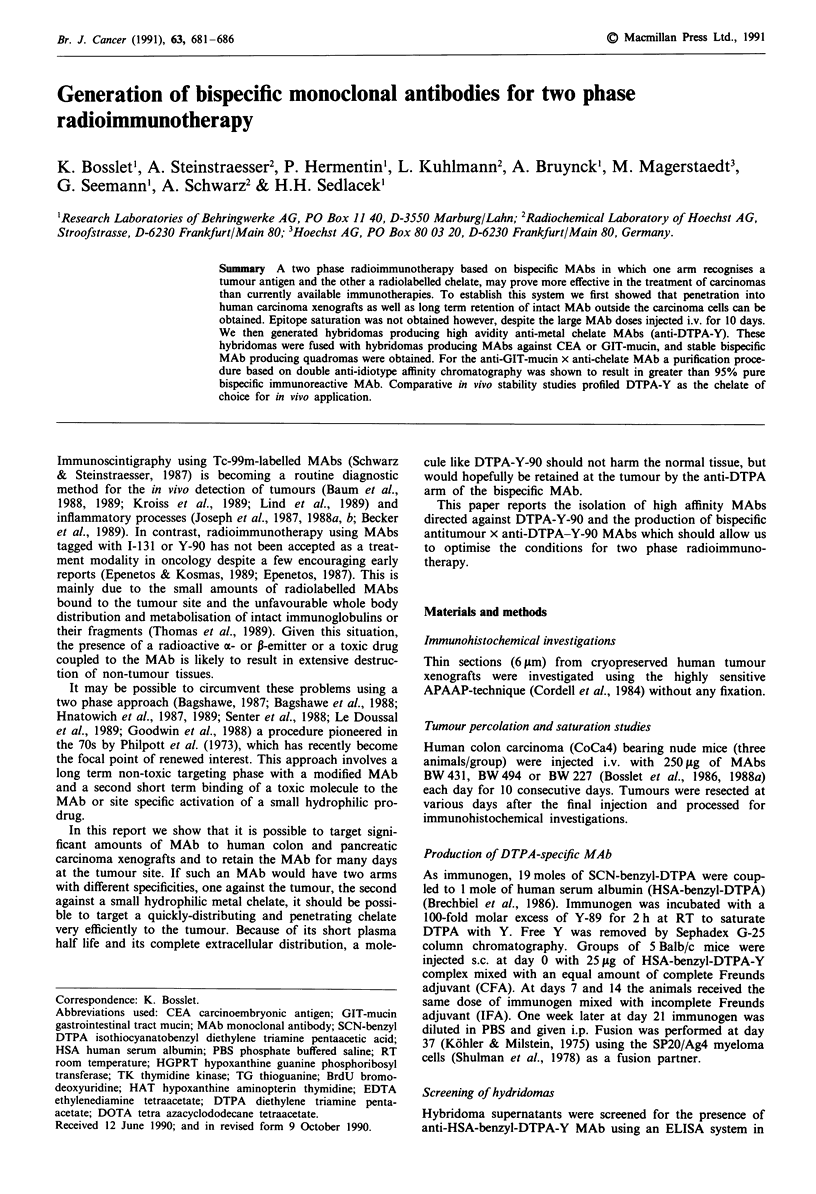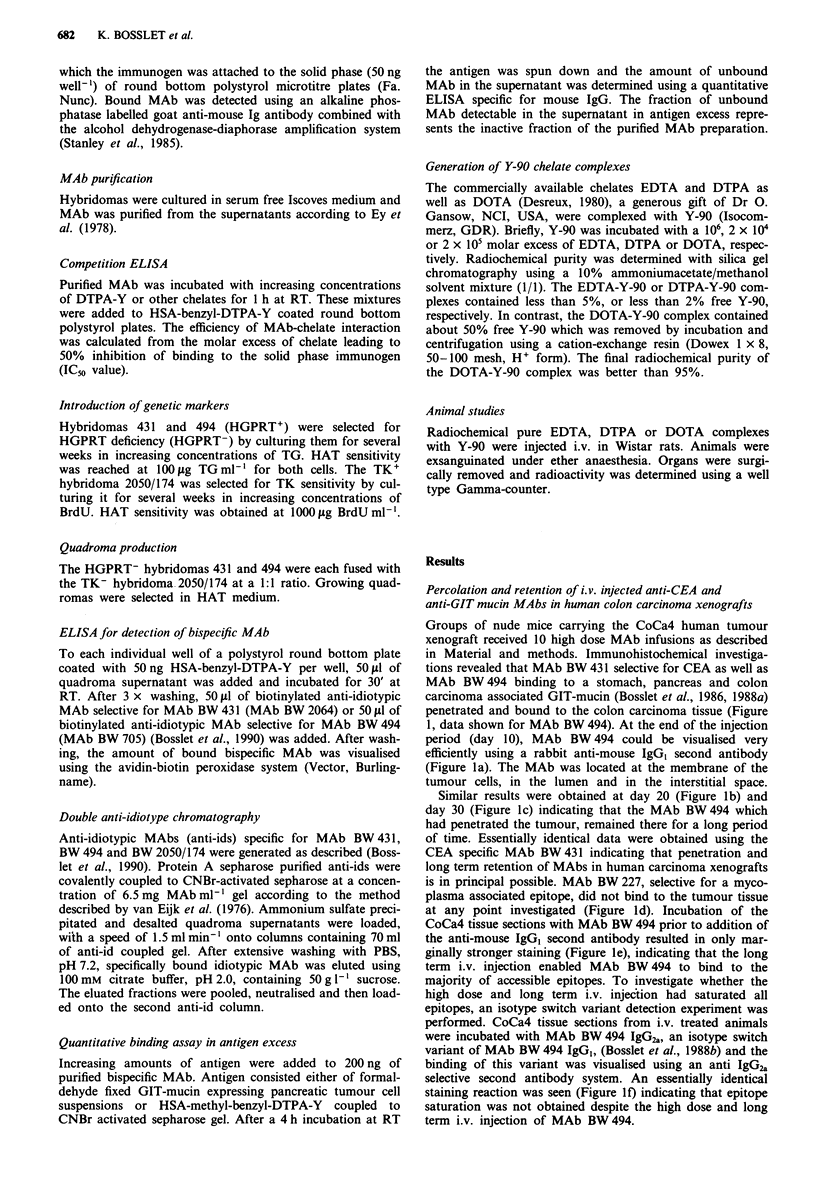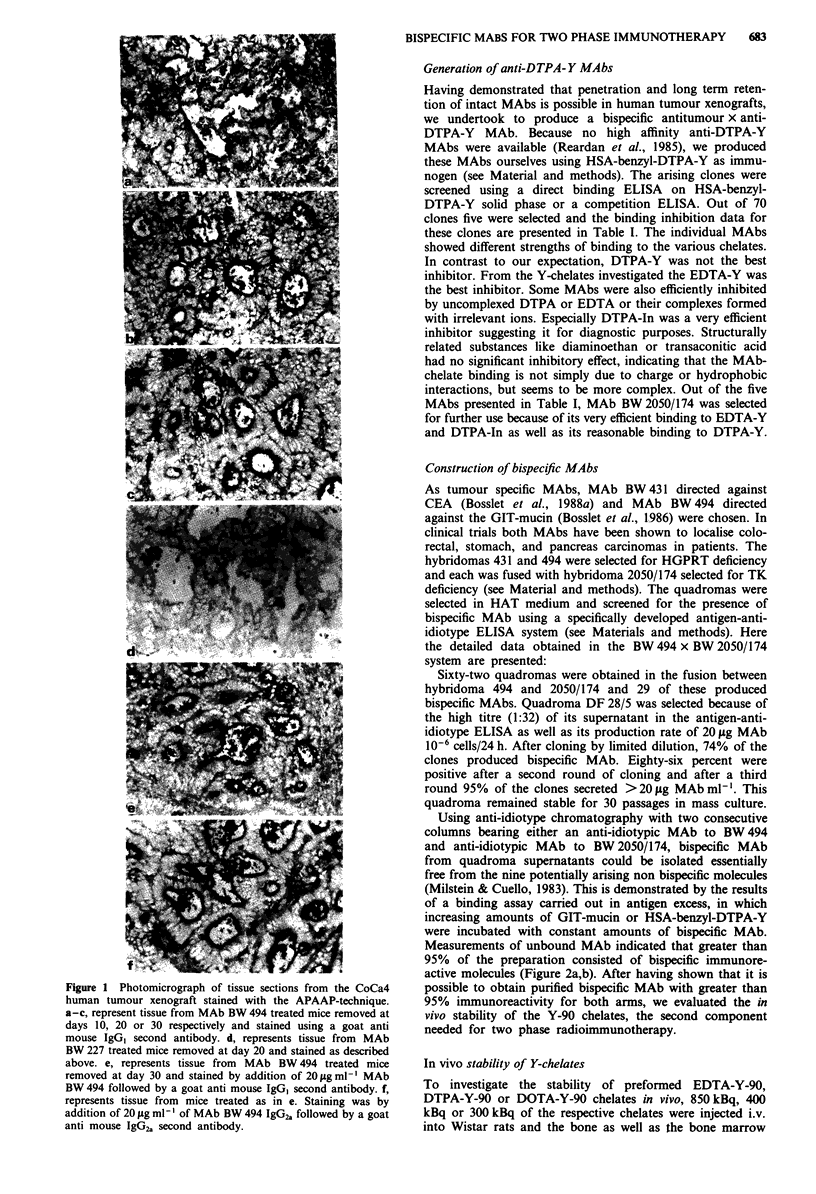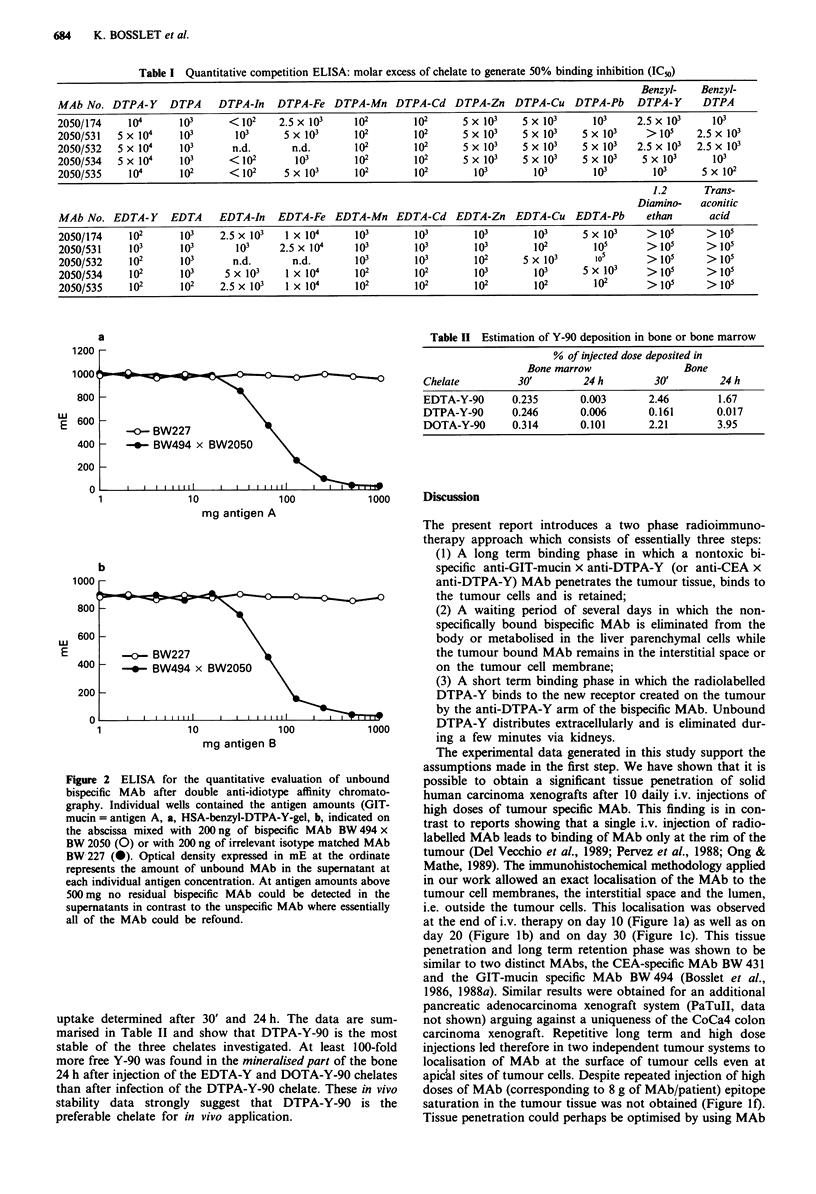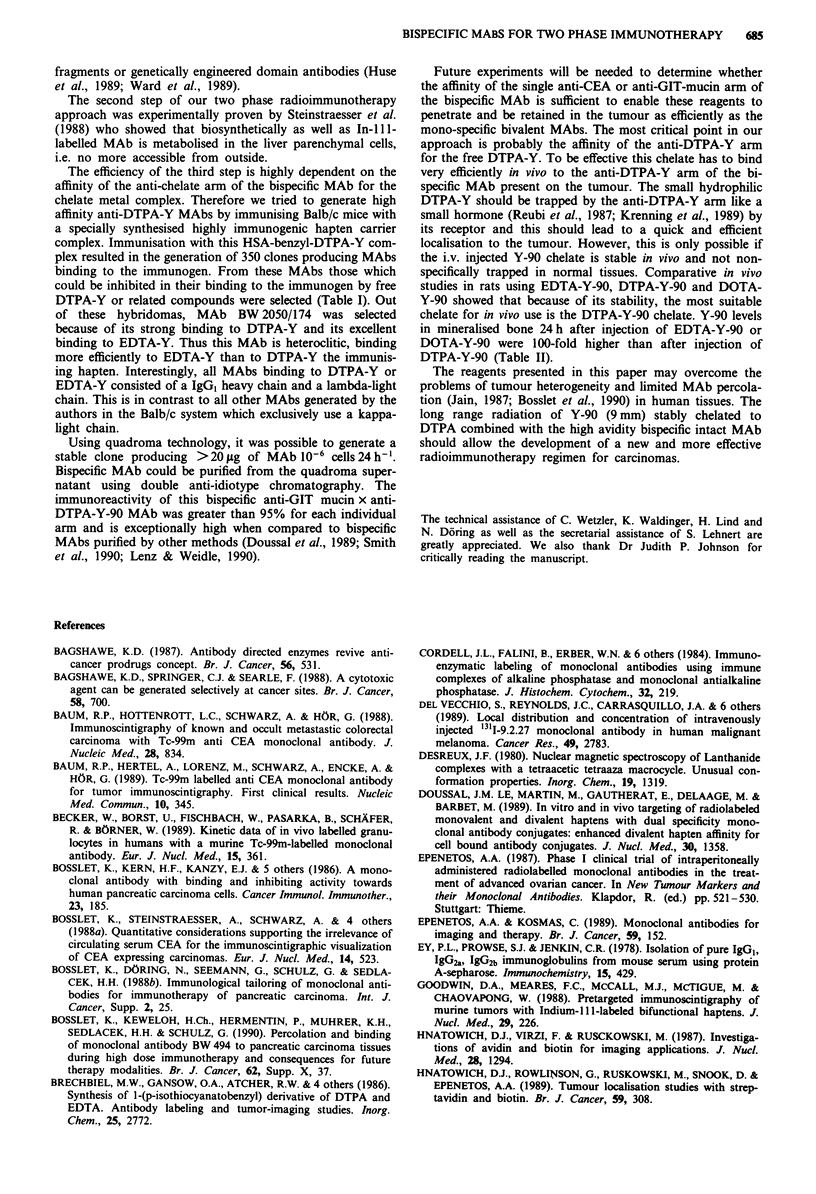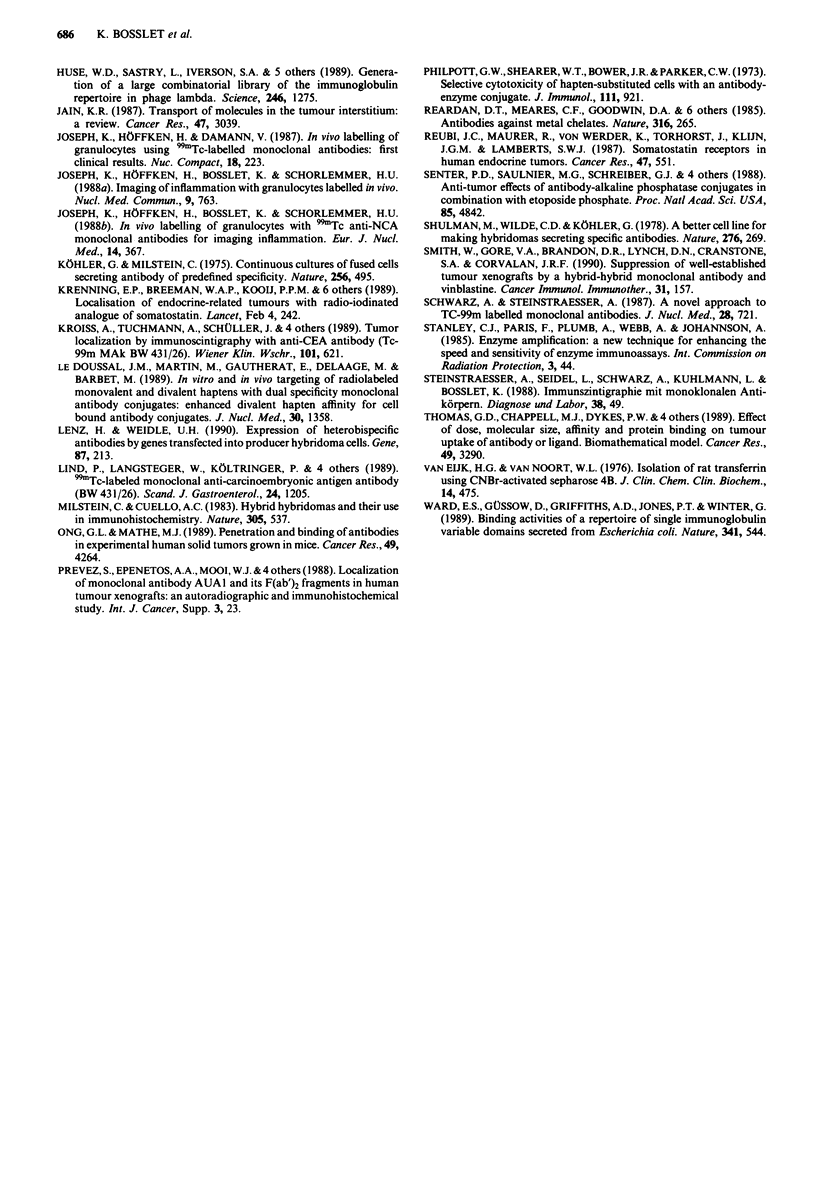# Generation of bispecific monoclonal antibodies for two phase radioimmunotherapy.

**DOI:** 10.1038/bjc.1991.155

**Published:** 1991-05

**Authors:** K. Bosslet, A. Steinstraesser, P. Hermentin, L. Kuhlmann, A. Bruynck, M. Magerstaedt, G. Seemann, A. Schwarz, H. H. Sedlacek

**Affiliations:** Research Laboratories of Behringwerke AG, Marburg/Lahn, Germany.

## Abstract

**Images:**


Br. J. Cancer (1991), 63, 681-686 © Macmillan Press Ltd., 1991

### Generation of bispecific monoclonal antibodies for two phase radioimmunotherapy

K. Bosslet[1], A. Steinstraesser[2], P. Hermentin[1], L. Kuhlmann[2], A. Bruynck[1], M. Magerstaedt[3], G. Seemann[1], A. Schwarz[2] & H.H. Sedlacek[1]

[1]*Research Laboratories of Behringwerke AG, PO Box 11 40, D-3550 Marburg/Lahn;* [2]*Radiochemical Laboratory of Hoechst AG, Stroofstrasse, D-6230 Frankfurt/Main 80;* [3]*Hoechst AG, PO Box 80 03 20, D-6230 Frankfurt/Main 80, Germany.*

**Summary** A two phase radioimmunotherapy based on bispecific MAbs in which one arm recognises a tumour antigen and the other a radiolabelled chelate, may prove more effective in the treatment of carcinomas than currently available immunotherapies. To establish this system we first showed that penetration into human carcinoma xenografts as well as long term retention of intact MAb outside the carcinoma cells can be obtained. Epitope saturation was not obtained however, despite the large MAb doses injected i.v. for 10 days. We then generated hybridomas producing high avidity anti-metal chelate MAbs (anti-DTPA-Y). These hybridomas were fused with hybridomas producing MAbs against CEA or GIT-mucin, and stable bispecific MAb producing quadromas were obtained. For the anti-GIT-mucin × anti-chelate MAb a purification procedure based on double anti-idiotype affinity chromatography was shown to result in greater than 95% pure bispecific immunoreactive MAb. Comparative *in vivo* stability studies profiled DTPA-Y as the chelate of choice for *in vivo* application.

Immunoscintigraphy using Tc-99m-labelled MAbs (Schwarz & Steinstraesser, 1987) is becoming a routine diagnostic method for the *in vivo* detection of tumours (Baum *et al.*, 1988, 1989; Kroiss *et al.*, 1989; Lind *et al.*, 1989) and inflammatory processes (Joseph *et al.*, 1987, 1988*a*, *b*; Becker *et al.*, 1989). In contrast, radioimmunotherapy using MAbs tagged with I-131 or Y-90 has not been accepted as a treatment modality in oncology despite a few encouraging early reports (Epenetos & Kosmas, 1989; Epenetos, 1987). This is mainly due to the small amounts of radiolabelled MAbs bound to the tumour site and the unfavourable whole body distribution and metabolisation of intact immunoglobulins or their fragments (Thomas *et al.*, 1989). Given this situation, the presence of a radioactive α- or β-emitter or a toxic drug coupled to the MAb is likely to result in extensive destruction of non-tumour tissues.

It may be possible to circumvent these problems using a two phase approach (Bagshawe, 1987; Bagshawe *et al.*, 1988; Hnatowich *et al.*, 1987, 1989; Senter *et al.*, 1988; Le Doussal *et al.*, 1989; Goodwin *et al.*, 1988) a procedure pioneered in the 70s by Philpott *et al.* (1973), which has recently become the focal point of renewed interest. This approach involves a long term non-toxic targeting phase with a modified MAb and a second short term binding of a toxic molecule to the MAb or site specific activation of a small hydrophilic prodrug.

In this report we show that it is possible to target significant amounts of MAb to human colon and pancreatic carcinoma xenografts and to retain the MAb for many days at the tumour site. If such an MAb would have two arms with different specificities, one against the tumour, the second against a small hydrophilic metal chelate, it should be possible to target a quickly-distributing and penetrating chelate very efficiently to the tumour. Because of its short plasma half life and its complete extracellular distribution, a molecule like DTPA-Y-90 should not harm the normal tissue, but would hopefully be retained at the tumour by the anti-DTPA arm of the bispecific MAb.

This paper reports the isolation of high affinity MAbs directed against DTPA-Y-90 and the production of bispecific antitumour × anti-DTPA-Y-90 MAbs which should allow us to optimise the conditions for two phase radioimmunotherapy.

### Materials and methods

### Immunohistochemical investigations

Thin sections (6 μm) from cryopreserved human tumour xenografts were investigated using the highly sensitive APAAP-technique (Cordell *et al.*, 1984) without any fixation.

### Tumour percolation and saturation studies

Human colon carcinoma (CoCa4) bearing nude mice (three animals/group) were injected i.v. with 250 μg of MAbs BW 431, BW 494 or BW 227 (Bosslet *et al.*, 1986, 1988*a*) each day for 10 consecutive days. Tumours were resected at various days after the final injection and processed for immunohistochemical investigations.

### Production of DTPA-specific MAb

As immunogen, 19 moles of SCN-benzyl-DTPA were coupled to 1 mole of human serum albumin (HSA-benzyl-DTPA) (Brechbiel *et al.*, 1986). Immunogen was incubated with a 100-fold molar excess of Y-89 for 2 h at RT to saturate DTPA with Y. Free Y was removed by Sephadex G-25 column chromatography. Groups of 5 Balb/c mice were injected s.c. at day 0 with 25 μg of HSA-benzyl-DTPA-Y complex mixed with an equal amount of complete Freunds adjuvant (CFA). At days 7 and 14 the animals received the same dose of immunogen mixed with incomplete Freunds adjuvant (IFA). One week later at day 21 immunogen was diluted in PBS and given i.p. Fusion was performed at day 37 (Köhler & Milstein, 1975) using the SP20/Ag4 myeloma cells (Shulman *et al.*, 1978) as a fusion partner.

### Screening of hydridomas

Hybridoma supernatants were screened for the presence of anti-HSA-benzyl-DTPA-Y MAb using an ELISA system in

Correspondence: K. Bosslet.

Abbreviations used: CEA carcinoembryonic antigen; GIT-mucin gastrointestinal tract mucin; MAb monoclonal antibody; SCN-benzyl DTPA isothiocyanatobenzyl diethylene triamine pentaacetic acid; HSA human serum albumin; PBS phosphate buffered saline; RT room temperature; HGPRT hypoxanthine guanine phosphoribosyl transferase; TK thymidine kinase; TG thioguanine; BrdU bromodeoxyuridine; HAT hypoxanthine aminopterin thymidine; EDTA ethylenediamine tetraacetate; DTPA diethylene triamine pentaacetate; DOTA tetra azacyclododecane tetraacetate.

Received 12 June 1990; and in revised form 9 October 1990.

which the immunogen was attached to the solid phase (50 ng well$^{-1}$) of round bottom polystyrol microtitre plates (Fa. Nunc). Bound MAb was detected using an alkaline phosphatase labelled goat anti-mouse Ig antibody combined with the alcohol dehydrogenase-diaphorase amplification system (Stanley *et al.*, 1985).

### *MAb purification*

Hybridomas were cultured in serum free Iscoves medium and MAb was purified from the supernatants according to Ey *et al.* (1978).

### *Competition ELISA*

Purified MAb was incubated with increasing concentrations of DTPA-Y or other chelates for 1 h at RT. These mixtures were added to HSA-benzyl-DTPA-Y coated round bottom polystyrol plates. The efficiency of MAb-chelate interaction was calculated from the molar excess of chelate leading to 50% inhibition of binding to the solid phase immunogen ($IC_{50}$ value).

### *Introduction of genetic markers*

Hybridomas 431 and 494 ($HGPRT^+$) were selected for HGPRT deficiency ($HGPRT^-$) by culturing them for several weeks in increasing concentrations of TG. HAT sensitivity was reached at 100 µg TG ml$^{-1}$ for both cells. The $TK^+$ hybridoma 2050/174 was selected for TK sensitivity by culturing it for several weeks in increasing concentrations of BrdU. HAT sensitivity was obtained at 1000 µg BrdU ml$^{-1}$.

### *Quadroma production*

The $HGPRT^-$ hybridomas 431 and 494 were each fused with the $TK^-$ hybridoma 2050/174 at a 1:1 ratio. Growing quadromas were selected in HAT medium.

### *ELISA for detection of bispecific MAb*

To each individual well of a polystyrol round bottom plate coated with 50 ng HSA-benzyl-DTPA-Y per well, 50 µl of quadroma supernatant was added and incubated for 30' at RT. After 3 × washing, 50 µl of biotinylated anti-idiotypic MAb selective for MAb BW 431 (MAb BW 2064) or 50 µl of biotinylated anti-idiotypic MAb selective for MAb BW 494 (MAb BW 705) (Bosslet *et al.*, 1990) was added. After washing, the amount of bound bispecific MAb was visualised using the avidin-biotin peroxidase system (Vector, Burlingname).

### *Double anti-idiotype chromatography*

Anti-idiotypic MAbs (anti-ids) specific for MAb BW 431, BW 494 and BW 2050/174 were generated as described (Bosslet *et al.*, 1990). Protein A sepharose purified anti-ids were covalently coupled to CNBr-activated sepharose at a concentration of 6.5 mg MAb ml$^{-1}$ gel according to the method described by van Eijk *et al.* (1976). Ammonium sulfate precipitated and desalted quadroma supernatants were loaded, with a speed of 1.5 ml min$^{-1}$ onto columns containing 70 ml of anti-id coupled gel. After extensive washing with PBS, pH 7.2, specifically bound idiotypic MAb was eluted using 100 mM citrate buffer, pH 2.0, containing 50 g l$^{-1}$ sucrose. The eluated fractions were pooled, neutralised and then loaded onto the second anti-id column.

### *Quantitative binding assay in antigen excess*

Increasing amounts of antigen were added to 200 ng of purified bispecific MAb. Antigen consisted either of formaldehyde fixed GIT-mucin expressing pancreatic tumour cell suspensions or HSA-methyl-benzyl-DTPA-Y coupled to CNBr activated sepharose gel. After a 4 h incubation at RT the antigen was spun down and the amount of unbound MAb in the supernatant was determined using a quantitative ELISA specific for mouse IgG. The fraction of unbound MAb detectable in the supernatant in antigen excess represents the inactive fraction of the purified MAb preparation.

### *Generation of Y-90 chelate complexes*

The commercially available chelates EDTA and DTPA as well as DOTA (Desreux, 1980), a generous gift of Dr O. Gansow, NCI, USA, were complexed with Y-90 (Isocommerz, GDR). Briefly, Y-90 was incubated with a $10^6$, $2 \times 10^4$ or $2 \times 10^5$ molar excess of EDTA, DTPA or DOTA, respectively. Radiochemical purity was determined with silica gel chromatography using a 10% ammoniumacetate/methanol solvent mixture (1/1). The EDTA-Y-90 or DTPA-Y-90 complexes contained less than 5%, or less than 2% free Y-90, respectively. In contrast, the DOTA-Y-90 complex contained about 50% free Y-90 which was removed by incubation and centrifugation using a cation-exchange resin (Dowex 1 × 8, 50–100 mesh, $H^+$ form). The final radiochemical purity of the DOTA-Y-90 complex was better than 95%.

### *Animal studies*

Radiochemical pure EDTA, DTPA or DOTA complexes with Y-90 were injected i.v. in Wistar rats. Animals were exsanguinated under ether anaesthesia. Organs were surgically removed and radioactivity was determined using a well type Gamma-counter.

### Results

### *Percolation and retention of i.v. injected anti-CEA and anti-GIT mucin MAbs in human colon carcinoma xenografts*

Groups of nude mice carrying the CoCa4 human tumour xenograft received 10 high dose MAb infusions as described in Material and methods. Immunohistochemical investigations revealed that MAb BW 431 selective for CEA as well as MAb BW 494 binding to a stomach, pancreas and colon carcinoma associated GIT-mucin (Bosslet *et al.*, 1986, 1988*a*) penetrated and bound to the colon carcinoma tissue (Figure 1, data shown for MAb BW 494). At the end of the injection period (day 10), MAb BW 494 could be visualised very efficiently using a rabbit anti-mouse $IgG_1$ second antibody (Figure 1a). The MAb was located at the membrane of the tumour cells, in the lumen and in the interstitial space.

Similar results were obtained at day 20 (Figure 1b) and day 30 (Figure 1c) indicating that the MAb BW 494 which had penetrated the tumour, remained there for a long period of time. Essentially identical data were obtained using the CEA specific MAb BW 431 indicating that penetration and long term retention of MAbs in human carcinoma xenografts is in principal possible. MAb BW 227, selective for a mycoplasma associated epitope, did not bind to the tumour tissue at any point investigated (Figure 1d). Incubation of the CoCa4 tissue sections with MAb BW 494 prior to addition of the anti-mouse $IgG_1$ second antibody resulted in only marginally stronger staining (Figure 1e), indicating that the long term i.v. injection enabled MAb BW 494 to bind to the majority of accessible epitopes. To investigate whether the high dose and long term i.v. injection had saturated all epitopes, an isotype switch variant detection experiment was performed. CoCa4 tissue sections from i.v. treated animals were incubated with MAb BW 494 $IgG_{2a}$, an isotype switch variant of MAb BW 494 $IgG_1$, (Bosslet *et al.*, 1988*b*) and the binding of this variant was visualised using an anti $IgG_{2a}$ selective second antibody system. An essentially identical staining reaction was seen (Figure 1f) indicating that epitope saturation was not obtained despite the high dose and long term i.v. injection of MAb BW 494.

**Figure 1** Photomicrograph of tissue sections from the CoCa4 human tumour xenograft stained with the APAAP-technique. **a–c**, represent tissue from MAb BW 494 treated mice removed at days 10, 20 or 30 respectively and stained using a goat anti mouse $IgG_1$ second antibody. **d**, represents tissue from MAb BW 227 treated mice removed at day 20 and stained as described above. **e**, represents tissue from MAb BW 494 treated mice removed at day 30 and stained by addition of 20 $\mu g\ ml^{-1}$ MAb BW 494 followed by a goat anti mouse $IgG_1$ second antibody. **f**, represents tissue from mice treated as in **e**. Staining was by addition of 20 $\mu g\ ml^{-1}$ of MAb BW 494 $IgG_{2a}$ followed by a goat anti mouse $IgG_{2a}$ second antibody.

*Generation of anti-DTPA-Y MAbs*

Having demonstrated that penetration and long term retention of intact MAbs is possible in human tumour xenografts, we undertook to produce a bispecific antitumour × anti-DTPA-Y MAb. Because no high affinity anti-DTPA-Y MAbs were available (Reardan *et al.*, 1985), we produced these MAbs ourselves using HSA-benzyl-DTPA-Y as immunogen (see Material and methods). The arising clones were screened using a direct binding ELISA on HSA-benzyl-DTPA-Y solid phase or a competition ELISA. Out of 70 clones five were selected and the binding inhibition data for these clones are presented in Table I. The individual MAbs showed different strengths of binding to the various chelates. In contrast to our expectation, DTPA-Y was not the best inhibitor. From the Y-chelates investigated the EDTA-Y was the best inhibitor. Some MAbs were also efficiently inhibited by uncomplexed DTPA or EDTA or their complexes formed with irrelevant ions. Especially DTPA-In was a very efficient inhibitor suggesting it for diagnostic purposes. Structurally related substances like diaminoethan or transaconitic acid had no significant inhibitory effect, indicating that the MAb-chelate binding is not simply due to charge or hydrophobic interactions, but seems to be more complex. Out of the five MAbs presented in Table I, MAb BW 2050/174 was selected for further use because of its very efficient binding to EDTA-Y and DTPA-In as well as its reasonable binding to DTPA-Y.

*Construction of bispecific MAbs*

As tumour specific MAbs, MAb BW 431 directed against CEA (Bosslet *et al.*, 1988*a*) and MAb BW 494 directed against the GIT-mucin (Bosslet *et al.*, 1986) were chosen. In clinical trials both MAbs have been shown to localise colorectal, stomach, and pancreas carcinomas in patients. The hybridomas 431 and 494 were selected for HGPRT deficiency and each was fused with hybridoma 2050/174 selected for TK deficiency (see Material and methods). The quadromas were selected in HAT medium and screened for the presence of bispecific MAb using a specifically developed antigen-anti-idiotype ELISA system (see Materials and methods). Here the detailed data obtained in the BW 494 × BW 2050/174 system are presented:

Sixty-two quadromas were obtained in the fusion between hybridoma 494 and 2050/174 and 29 of these produced bispecific MAbs. Quadroma DF 28/5 was selected because of the high titre (1:32) of its supernatant in the antigen-anti-idiotype ELISA as well as its production rate of 20 $\mu g$ MAb $10^{-6}$ cells/24 h. After cloning by limited dilution, 74% of the clones produced bispecific MAb. Eighty-six percent were positive after a second round of cloning and after a third round 95% of the clones secreted $>20\ \mu g$ MAb $ml^{-1}$. This quadroma remained stable for 30 passages in mass culture.

Using anti-idiotype chromatography with two consecutive columns bearing either an anti-idiotypic MAb to BW 494 and anti-idiotypic MAb to BW 2050/174, bispecific MAb from quadroma supernatants could be isolated essentially free from the nine potentially arising non bispecific molecules (Milstein & Cuello, 1983). This is demonstrated by the results of a binding assay carried out in antigen excess, in which increasing amounts of GIT-mucin or HSA-benzyl-DTPA-Y were incubated with constant amounts of bispecific MAb. Measurements of unbound MAb indicated that greater than 95% of the preparation consisted of bispecific immunoreactive molecules (Figure 2a,b). After having shown that it is possible to obtain purified bispecific MAb with greater than 95% immunoreactivity for both arms, we evaluated the *in vivo* stability of the Y-90 chelates, the second component needed for two phase radioimmunotherapy.

In vivo *stability of Y-chelates*

To investigate the stability of preformed EDTA-Y-90, DTPA-Y-90 or DOTA-Y-90 chelates *in vivo*, 850 kBq, 400 kBq or 300 kBq of the respective chelates were injected i.v. into Wistar rats and the bone as well as the bone marrow

**Table I** Quantitative competition ELISA: molar excess of chelate to generate 50% binding inhibition ($IC_{50}$)

| *MAb No.* | *DTPA-Y* | *DTPA* | *DTPA-In* | *DTPA-Fe* | *DTPA-Mn* | *DTPA-Cd* | *DTPA-Zn* | *DTPA-Cu* | *DTPA-Pb* | *Benzyl-DTPA-Y* | *Benzyl-DTPA* |
|---|---|---|---|---|---|---|---|---|---|---|---|
| 2050/174 | $10^4$ | $10^3$ | $<10^2$ | $2.5 \times 10^3$ | $10^2$ | $10^2$ | $5 \times 10^3$ | $5 \times 10^3$ | $10^3$ | $2.5 \times 10^3$ | $10^3$ |
| 2050/531 | $5 \times 10^4$ | $10^3$ | $10^3$ | $5 \times 10^3$ | $10^2$ | $10^2$ | $5 \times 10^3$ | $5 \times 10^3$ | $5 \times 10^3$ | $>10^5$ | $2.5 \times 10^3$ |
| 2050/532 | $5 \times 10^4$ | $10^3$ | n.d. | n.d. | $10^2$ | $10^2$ | $5 \times 10^3$ | $5 \times 10^3$ | $5 \times 10^3$ | $2.5 \times 10^3$ | $2.5 \times 10^3$ |
| 2050/534 | $5 \times 10^4$ | $10^3$ | $<10^2$ | $10^3$ | $10^2$ | $10^2$ | $5 \times 10^3$ | $5 \times 10^3$ | $5 \times 10^3$ | $5 \times 10^3$ | $10^3$ |
| 2050/535 | $10^4$ | $10^2$ | $<10^2$ | $5 \times 10^3$ | $10^2$ | $10^2$ | $10^3$ | $10^3$ | $10^3$ | $10^3$ | $5 \times 10^2$ |
| *MAb No.* | *EDTA-Y* | *EDTA* | *EDTA-In* | *EDTA-Fe* | *EDTA-Mn* | *EDTA-Cd* | *EDTA-Zn* | *EDTA-Cu* | *EDTA-Pb* | *1.2 Diamino-ethan* | *Trans-aconitic acid* |
| 2050/174 | $10^2$ | $10^3$ | $2.5 \times 10^3$ | $1 \times 10^4$ | $10^3$ | $10^3$ | $10^3$ | $10^3$ | $5 \times 10^3$ | $>10^5$ | $>10^5$ |
| 2050/531 | $10^3$ | $10^3$ | $10^3$ | $2.5 \times 10^4$ | $10^3$ | $10^3$ | $10^3$ | $10^2$ | $10^5$ | $>10^5$ | $>10^5$ |
| 2050/532 | $10^2$ | $10^3$ | n.d. | n.d. | $10^3$ | $10^3$ | $10^2$ | $5 \times 10^3$ | $10^5$ | $>10^5$ | $>10^5$ |
| 2050/534 | $10^2$ | $10^3$ | $5 \times 10^3$ | $1 \times 10^4$ | $10^2$ | $10^2$ | $10^3$ | $10^3$ | $5 \times 10^3$ | $>10^5$ | $>10^5$ |
| 2050/535 | $10^2$ | $10^2$ | $2.5 \times 10^3$ | $1 \times 10^4$ | $10^2$ | $10^2$ | $10^2$ | $10^2$ | $10^2$ | $>10^5$ | $>10^5$ |

**Figure 2** ELISA for the quantitative evaluation of unbound bispecific MAb after double anti-idiotype affinity chromatography. Individual wells contained the antigen amounts (GIT-mucin = antigen A, **a**, HSA-benzyl-DTPA-Y-gel, **b**, indicated on the abscissa mixed with 200 ng of bispecific MAb BW 494 × BW 2050 (○) or with 200 ng of irrelevant isotype matched MAb BW 227 (●). Optical density expressed in mE at the ordinate represents the amount of unbound MAb in the supernatant at each individual antigen concentration. At antigen amounts above 500 mg no residual bispecific MAb could be detected in the supernatants in contrast to the unspecific MAb where essentially all of the MAb could be refound.

uptake determined after 30′ and 24 h. The data are summarised in Table II and show that DTPA-Y-90 is the most stable of the three chelates investigated. At least 100-fold more free Y-90 was found in the *mineralised part* of the bone 24 h after injection of the EDTA-Y and DOTA-Y-90 chelates than after infection of the DTPA-Y-90 chelate. These *in vivo* stability data strongly suggest that DTPA-Y-90 is the preferable chelate for *in vivo* application.

**Table II** Estimation of Y-90 deposition in bone or bone marrow

| | *% of injected dose deposited in* | | | |
|---|---|---|---|---|
| | *Bone marrow* | | *Bone* | |
| *Chelate* | *30′* | *24 h* | *30′* | *24 h* |
| EDTA-Y-90 | 0.235 | 0.003 | 2.46 | 1.67 |
| DTPA-Y-90 | 0.246 | 0.006 | 0.161 | 0.017 |
| DOTA-Y-90 | 0.314 | 0.101 | 2.21 | 3.95 |

### Discussion

The present report introduces a two phase radioimmunotherapy approach which consists of essentially three steps:

(1) A long term binding phase in which a nontoxic bispecific anti-GIT-mucin × anti-DTPA-Y (or anti-CEA × anti-DTPA-Y) MAb penetrates the tumour tissue, binds to the tumour cells and is retained;

(2) A waiting period of several days in which the non-specifically bound bispecific MAb is eliminated from the body or metabolised in the liver parenchymal cells while the tumour bound MAb remains in the interstitial space or on the tumour cell membrane;

(3) A short term binding phase in which the radiolabelled DTPA-Y binds to the new receptor created on the tumour by the anti-DTPA-Y arm of the bispecific MAb. Unbound DTPA-Y distributes extracellularly and is eliminated during a few minutes via kidneys.

The experimental data generated in this study support the assumptions made in the first step. We have shown that it is possible to obtain a significant tissue penetration of solid human carcinoma xenografts after 10 daily i.v. injections of high doses of tumour specific MAb. This finding is in contrast to reports showing that a single i.v. injection of radiolabelled MAb leads to binding of MAb only at the rim of the tumour (Del Vecchio *et al.*, 1989; Pervez *et al.*, 1988; Ong & Mathe, 1989). The immunohistochemical methodology applied in our work allowed an exact localisation of the MAb to the tumour cell membranes, the interstitial space and the lumen, i.e. outside the tumour cells. This localisation was observed at the end of i.v. therapy on day 10 (Figure 1a) as well as on day 20 (Figure 1b) and on day 30 (Figure 1c). This tissue penetration and long term retention phase was shown to be similar to two distinct MAbs, the CEA-specific MAb BW 431 and the GIT-mucin specific MAb BW 494 (Bosslet *et al.*, 1986, 1988*a*). Similar results were obtained for an additional pancreatic adenocarcinoma xenograft system (PaTuII, data not shown) arguing against a uniqueness of the CoCa4 colon carcinoma xenograft. Repetitive long term and high dose injections led therefore in two independent tumour systems to localisation of MAb at the surface of tumour cells even at apical sites of tumour cells. Despite repeated injection of high doses of MAb (corresponding to 8 g of MAb/patient) epitope saturation in the tumour tissue was not obtained (Figure 1f). Tissue penetration could perhaps be optimised by using MAb fragments or genetically engineered domain antibodies (Huse *et al.*, 1989; Ward *et al.*, 1989).

The second step of our two phase radioimmunotherapy approach was experimentally proven by Steinstraesser *et al.* (1988) who showed that biosynthetically as well as In-111-labelled MAb is metabolised in the liver parenchymal cells, i.e. no more accessible from outside.

The efficiency of the third step is highly dependent on the affinity of the anti-chelate arm of the bispecific MAb for the chelate metal complex. Therefore we tried to generate high affinity anti-DTPA-Y MAbs by immunising Balb/c mice with a specially synthesised highly immunogenic hapten carrier complex. Immunisation with this HSA-benzyl-DTPA-Y complex resulted in the generation of 350 clones producing MAbs binding to the immunogen. From these MAbs those which could be inhibited in their binding to the immunogen by free DTPA-Y or related compounds were selected (Table I). Out of these hybridomas, MAb BW 2050/174 was selected because of its strong binding to DTPA-Y and its excellent binding to EDTA-Y. Thus this MAb is heteroclitic, binding more efficiently to EDTA-Y than to DTPA-Y the immunising hapten. Interestingly, all MAbs binding to DTPA-Y or EDTA-Y consisted of a $IgG_1$ heavy chain and a lambda-light chain. This is in contrast to all other MAbs generated by the authors in the Balb/c system which exclusively use a kappa-light chain.

Using quadroma technology, it was possible to generate a stable clone producing $>20\,\mu g$ of MAb $10^{-6}$ cells $24\,h^{-1}$. Bispecific MAb could be purified from the quadroma supernatant using double anti-idiotype chromatography. The immunoreactivity of this bispecific anti-GIT mucin × anti-DTPA-Y-90 MAb was greater than 95% for each individual arm and is exceptionally high when compared to bispecific MAbs purified by other methods (Doussal *et al.*, 1989; Smith *et al.*, 1990; Lenz & Weidle, 1990).

Future experiments will be needed to determine whether the affinity of the single anti-CEA or anti-GIT-mucin arm of the bispecific MAb is sufficient to enable these reagents to penetrate and be retained in the tumour as efficiently as the mono-specific bivalent MAbs. The most critical point in our approach is probably the affinity of the anti-DTPA-Y arm for the free DTPA-Y. To be effective this chelate has to bind very efficiently *in vivo* to the anti-DTPA-Y arm of the bispecific MAb present on the tumour. The small hydrophilic DTPA-Y should be trapped by the anti-DTPA-Y arm like a small hormone (Reubi *et al.*, 1987; Krenning *et al.*, 1989) by its receptor and this should lead to a quick and efficient localisation to the tumour. However, this is only possible if the i.v. injected Y-90 chelate is stable *in vivo* and not non-specifically trapped in normal tissues. Comparative *in vivo* studies in rats using EDTA-Y-90, DTPA-Y-90 and DOTA-Y-90 showed that because of its stability, the most suitable chelate for *in vivo* use is the DTPA-Y-90 chelate. Y-90 levels in mineralised bone 24 h after injection of EDTA-Y-90 or DOTA-Y-90 were 100-fold higher than after injection of DTPA-Y-90 (Table II).

The reagents presented in this paper may overcome the problems of tumour heterogeneity and limited MAb percolation (Jain, 1987; Bosslet *et al.*, 1990) in human tissues. The long range radiation of Y-90 (9 mm) stably chelated to DTPA combined with the high avidity bispecific intact MAb should allow the development of a new and more effective radioimmunotherapy regimen for carcinomas.

The technical assistance of C. Wetzler, K. Waldinger, H. Lind and N. Döring as well as the secretarial assistance of S. Lehnert are greatly appreciated. We also thank Dr Judith P. Johnson for critically reading the manuscript.

### References

BAGSHAWE, K.D. (1987). Antibody directed enzymes revive anti-cancer prodrugs concept. *Br. J. Cancer*, **56**, 531.

BAGSHAWE, K.D., SPRINGER, C.J. & SEARLE, F. (1988). A cytotoxic agent can be generated selectively at cancer sites. *Br. J. Cancer*, **58**, 700.

BAUM, R.P., HOTTENROTT, L.C., SCHWARZ, A. & HÖR, G. (1988). Immunoscintigraphy of known and occult metastastic colorectal carcinoma with Tc-99m anti CEA monoclonal antibody. *J. Nucleic Med.*, **28**, 834.

BAUM, R.P., HERTEL, A., LORENZ, M., SCHWARZ, A., ENCKE, A. & HÖR, G. (1989). Tc-99m labelled anti CEA monoclonal antibody for tumor immunoscintigraphy. First clinical results. *Nucleic Med. Commun.*, **10**, 345.

BECKER, W., BORST, U., FISCHBACH, W., PASARKA, B., SCHÄFER, R. & BÖRNER, W. (1989). Kinetic data of in vivo labelled granulocytes in humans with a murine Tc-99m-labelled monoclonal antibody. *Eur. J. Nucl. Med.*, **15**, 361.

BOSSLET, K., KERN, H.F., KANZY, E.J. & 5 others (1986). A monoclonal antibody with binding and inhibiting activity towards human pancreatic carcinoma cells. *Cancer Immunol. Immunother.*, **23**, 185.

BOSSLET, K., STEINSTRAESSER, A., SCHWARZ, A. & 4 others (1988*a*). Quantitative considerations supporting the irrelevance of circulating serum CEA for the immunoscintigraphic visualization of CEA expressing carcinomas. *Eur. J. Nucl. Med.*, **14**, 523.

BOSSLET, K., DÖRING, N., SEEMANN, G., SCHULZ, G. & SEDLACEK, H.H. (1988*b*). Immunological tailoring of monoclonal antibodies for immunotherapy of pancreatic carcinoma. *Int. J. Cancer*, Supp. **2**, 25.

BOSSLET, K., KEWELOH, H.Ch., HERMENTIN, P., MUHRER, K.H., SEDLACEK, H.H. & SCHULZ, G. (1990). Percolation and binding of monoclonal antibody BW 494 to pancreatic carcinoma tissues during high dose immunotherapy and consequences for future therapy modalities. *Br. J. Cancer*, **62**, Supp. X, 37.

BRECHBIEL, M.W., GANSOW, O.A., ATCHER, R.W. & 4 others (1986). Synthesis of 1-(p-isothiocyanatobenzyl) derivative of DTPA and EDTA. Antibody labeling and tumor-imaging studies. *Inorg. Chem.*, **25**, 2772.

CORDELL, J.L., FALINI, B., ERBER, W.N. & 6 others (1984). Immunoenzymatic labeling of monoclonal antibodies using immune complexes of alkaline phosphatase and monoclonal antialkaline phosphatase. *J. Histochem. Cytochem.*, **32**, 219.

DEL VECCHIO, S., REYNOLDS, J.C., CARRASQUILLO, J.A. & 6 others (1989). Local distribution and concentration of intravenously injected $^{131}$I-9.2.27 monoclonal antibody in human malignant melanoma. *Cancer Res.*, **49**, 2783.

DESREUX, J.F. (1980). Nuclear magnetic spectroscopy of Lanthanide complexes with a tetraacetic tetraaza macrocycle. Unusual conformation properties. *Inorg. Chem.*, **19**, 1319.

DOUSSAL, J.M. LE, MARTIN, M., GAUTHERAT, E., DELAAGE, M. & BARBET, M. (1989). In vitro and in vivo targeting of radiolabeled monovalent and divalent haptens with dual specificity monoclonal antibody conjugates: enhanced divalent hapten affinity for cell bound antibody conjugates. *J. Nucl. Med.*, **30**, 1358.

EPENETOS, A.A. (1987). Phase I clinical trial of intraperitoneally administered radiolabelled monoclonal antibodies in the treatment of advanced ovarian cancer. In *New Tumour Markers and their Monoclonal Antibodies*. Klapdor, R. (ed.) pp. 521–530. Stuttgart: Thieme.

EPENETOS, A.A. & KOSMAS, C. (1989). Monoclonal antibodies for imaging and therapy. *Br. J. Cancer*, **59**, 152.

EY, P.L., PROWSE, S.J. & JENKIN, C.R. (1978). Isolation of pure $IgG_1$, $IgG_{2a}$, $IgG_{2b}$ immunoglobulins from mouse serum using protein A-sepharose. *Immunochemistry*, **15**, 429.

GOODWIN, D.A., MEARES, F.C., MCCALL, M.J., MCTIGUE, M. & CHAOVAPONG, W. (1988). Pretargeted immunoscintigraphy of murine tumors with Indium-111-labeled bifunctional haptens. *J. Nucl. Med.*, **29**, 226.

HNATOWICH, D.J., VIRZI, F. & RUSCKOWSKI, M. (1987). Investigations of avidin and biotin for imaging applications. *J. Nucl. Med.*, **28**, 1294.

HNATOWICH, D.J., ROWLINSON, G., RUSKOWSKI, M., SNOOK, D. & EPENETOS, A.A. (1989). Tumour localisation studies with streptavidin and biotin. *Br. J. Cancer*, **59**, 308.

HUSE, W.D., SASTRY, L., IVERSON, S.A. & 5 others (1989). Generation of a large combinatorial library of the immunoglobulin repertoire in phage lambda. *Science*, **246**, 1275.

JAIN, K.R. (1987). Transport of molecules in the tumour interstitium: a review. *Cancer Res.*, **47**, 3039.

JOSEPH, K., HÖFFKEN, H. & DAMANN, V. (1987). *In vivo* labelling of granulocytes using $^{99m}$Tc-labelled monoclonal antibodies: first clinical results. *Nuc. Compact*, **18**, 223.

JOSEPH, K., HÖFFKEN, H., BOSSLET, K. & SCHORLEMMER, H.U. (1988*a*). Imaging of inflammation with granulocytes labelled *in vivo*. *Nucl. Med. Commun.*, **9**, 763.

JOSEPH, K., HÖFFKEN, H., BOSSLET, K. & SCHORLEMMER, H.U. (1988*b*). *In vivo* labelling of granulocytes with $^{99m}$Tc anti-NCA monoclonal antibodies for imaging inflammation. *Eur. J. Nucl. Med.*, **14**, 367.

KÖHLER, G. & MILSTEIN, C. (1975). Continuous cultures of fused cells secreting antibody of predefined specificity. *Nature*, **256**, 495.

KRENNING, E.P., BREEMAN, W.A.P., KOOIJ, P.P.M. & 6 others (1989). Localisation of endocrine-related tumours with radio-iodinated analogue of somatostatin. *Lancet*, Feb 4, 242.

KROISS, A., TUCHMANN, A., SCHÜLLER, J. & 4 others (1989). Tumor localization by immunoscintigraphy with anti-CEA antibody (Tc-99m MAk BW 431/26). *Wiener Klin. Wschr.*, **101**, 621.

LE DOUSSAL, J.M., MARTIN, M., GAUTHERAT, E., DELAAGE, M. & BARBET, M. (1989). *In vitro* and *in vivo* targeting of radiolabeled monovalent and divalent haptens with dual specificity monoclonal antibody conjugates: enhanced divalent hapten affinity for cell bound antibody conjugates. *J. Nucl. Med.*, **30**, 1358.

LENZ, H. & WEIDLE, U.H. (1990). Expression of heterobispecific antibodies by genes transfected into producer hybridoma cells. *Gene*, **87**, 213.

LIND, P., LANGSTEGER, W., KÖLTRINGER, P. & 4 others (1989). $^{99m}$Tc-labeled monoclonal anti-carcinoembryonic antigen antibody (BW 431/26). *Scand. J. Gastroenterol.*, **24**, 1205.

MILSTEIN, C. & CUELLO, A.C. (1983). Hybrid hybridomas and their use in immunohistochemistry. *Nature*, **305**, 537.

ONG, G.L. & MATHE, M.J. (1989). Penetration and binding of antibodies in experimental human solid tumors grown in mice. *Cancer Res.*, **49**, 4264.

PREVEZ, S., EPENETOS, A.A., MOOI, W.J. & 4 others (1988). Localization of monoclonal antibody AUA1 and its F(ab')$_2$ fragments in human tumour xenografts: an autoradiographic and immunohistochemical study. *Int. J. Cancer*, Supp. 3, 23.

PHILPOTT, G.W., SHEARER, W.T., BOWER, J.R. & PARKER, C.W. (1973). Selective cytotoxicity of hapten-substituted cells with an antibody-enzyme conjugate. *J. Immunol.*, **111**, 921.

REARDAN, D.T., MEARES, C.F., GOODWIN, D.A. & 6 others (1985). Antibodies against metal chelates. *Nature*, **316**, 265.

REUBI, J.C., MAURER, R., VON WERDER, K., TORHORST, J., KLIJN, J.G.M. & LAMBERTS, S.W.J. (1987). Somatostatin receptors in human endocrine tumors. *Cancer Res.*, **47**, 551.

SENTER, P.D., SAULNIER, M.G., SCHREIBER, G.J. & 4 others (1988). Anti-tumor effects of antibody-alkaline phosphatase conjugates in combination with etoposide phosphate. *Proc. Natl Acad. Sci. USA*, **85**, 4842.

SHULMAN, M., WILDE, C.D. & KÖHLER, G. (1978). A better cell line for making hybridomas secreting specific antibodies. *Nature*, **276**, 269.

SMITH, W., GORE, V.A., BRANDON, D.R., LYNCH, D.N., CRANSTONE, S.A. & CORVALAN, J.R.F. (1990). Suppression of well-established tumour xenografts by a hybrid-hybrid monoclonal antibody and vinblastine. *Cancer Immunol. Immunother.*, **31**, 157.

SCHWARZ, A. & STEINSTRAESSER, A. (1987). A novel approach to TC-99m labelled monoclonal antibodies. *J. Nucl. Med.*, **28**, 721.

STANLEY, C.J., PARIS, F., PLUMB, A., WEBB, A. & JOHANNSON, A. (1985). Enzyme amplification: a new technique for enhancing the speed and sensitivity of enzyme immunoassays. *Int. Commission on Radiation Protection*, **3**, 44.

STEINSTRAESSER, A., SEIDEL, L., SCHWARZ, A., KUHLMANN, L. & BOSSLET, K. (1988). Immunszintigraphie mit monoklonalen Antikörpern. *Diagnose und Labor*, **38**, 49.

THOMAS, G.D., CHAPPELL, M.J., DYKES, P.W. & 4 others (1989). Effect of dose, molecular size, affinity and protein binding on tumour uptake of antibody or ligand. Biomathematical model. *Cancer Res.*, **49**, 3290.

VAN EIJK, H.G. & VAN NOORT, W.L. (1976). Isolation of rat transferrin using CNBr-activated sepharose 4B. *J. Clin. Chem. Clin. Biochem.*, **14**, 475.

WARD, E.S., GÜSSOW, D., GRIFFITHS, A.D., JONES, P.T. & WINTER, G. (1989). Binding activities of a repertoire of single immunoglobulin variable domains secreted from *Escherichia coli*. *Nature*, **341**, 544.